# Hepatitis B-associated cirrhosis combined with multiple nodular fatty infiltrates of the liver: A rare benign disease misdiagnosed as hepatocellular carcinoma – a case report

**DOI:** 10.1097/MD.0000000000045958

**Published:** 2026-05-12

**Authors:** Jiayin Zhao, Jian Wang, Yuqi Wang, Wei Zhang, Wei Sang

**Affiliations:** a Department of Pathology, The First Affiliated Hospital of Xinjiang Medical University, Urumqi, Xinjiang, P.R. China; b Department of Imaging, The First Affiliated Hospital of Xinjiang Medical University, Urumqi, Xinjiang, P.R. China.

**Keywords:** case report, cirrhosis, multifocal nodular fatty infiltration, pathologic diagnosis, viral hepatitis B

## Abstract

**Rationale:**

Chronic hepatitis B virus infection, if not treated with standard therapies, usually progresses to cirrhosis, which is a major risk factor for hepatocellular carcinoma (HCC). Steatosis can lead to fatty liver disease; if hepatic fatty infiltration shows multinodular changes, it is called hepatic multinodular fatty infiltration (MNFIL). However, this pattern of fatty infiltration is rare. Cirrhosis combined with MNFIL is very rare and easily misdiagnosed as HCC.

**Patient concerns:**

The patient was a 70-year-old woman with a 22-year history of hepatitis B. Physical examination revealed multiple masses in the liver. After admission, the computed tomography, ultrasound examination, and hepatic angiography findings were inconsistent.

**Diagnoses:**

Hepatitis B-associated cirrhosis combined with multiple nodular fatty liver infiltrates.

**Interventions:**

The patient ultimately underwent a partial resection of the liver mass.

**Outcomes:**

The patient was followed-up for 5 months and is currently doing well.

**Lessons:**

The appearance of multiple nodules against the background of cirrhosis does not necessarily indicate malignant nodules. If this awareness is considered during the diagnostic process, misdiagnoses can be avoided.

## 1. Introduction

Chronic hepatitis B virus infection, if not treated with standard therapies, usually progresses to cirrhosis, which is a major risk factor for hepatocellular carcinoma (HCC).^[1]^ Steatosis can lead to fatty liver disease; if hepatic fatty infiltration shows multinodular changes, it is called hepatic multinodular fatty infiltration (MNFIL). This pattern of fatty infiltration is rare and is easily misdiagnosed as a primary or metastatic tumor of the liver.^[2–4]^ Cirrhosis combined with MNFIL is very rare and easily misdiagnosed as HCC. This is the first report of a case of hepatitis B cirrhosis combined with MNFIL, which provides a reference for avoiding misdiagnosis and overtreatment by understanding its pathogenesis and clinical significance.

## 2. Case report

### 2.1. Basic information

A 70-year-old female, was admitted to the hospital with multiple masses in the liver on physical examination. She had a 22-year history of viral hepatitis B that was not treated regularly. She had a 10-year history of hypertension and was taking medication regularly; however, her blood pressure was not well controlled (154/94 mm Hg). There had no history of alcohol consumption or diabetes mellitus. vital signs were stable, except for elevated blood pressure, and there were no abnormal abdominal signs. This study was approved by the Institutional Review Board of the First Affiliated Hospital of Xinjiang Medical University Foundation Institutional Review Board (K202503-30). Written informed consent was obtained from all patients for information collection.

### 2.2. Imaging examination

**Figure 1.**
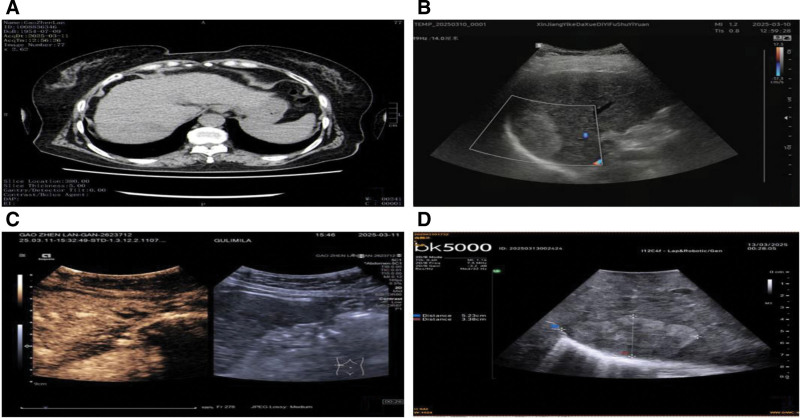
Radiographic examination. (A) Abdominal CT reveals liver cirrhosis, with a 7.25 × 4.96 cm oval mixed slightly hypodense lesion in the right lobe, of undetermined nature. (B) Abdominal ultrasound shows liver cirrhosis, with oval lesions measuring 5.5 × 3.7 × 5.6 cm in segment S7 and 2.2 × 2.0 × 1.8 cm at the inferior margin of the left lobe, suggesting a tendency towards malignancy; Multiple mid-to-high echogenic nodules are observed in the remaining liver, the largest measuring 1.0 × 0.9 cm. (C) Liver angiography indicates cirrhosis, with lesions in segment S7 (5.6 × 5.5 × 3.7 cm) and the left lobe nodule (2.2 × 2.0 × 1.8 cm) showing no significant blood flow signals, suggesting atypical hemangiomas; Multiple mid-to-high echogenic proliferative nodules are observed in the remaining liver. (D) Intraoperative ultrasound shows that the S7 segment lesion is likely benign. CT = computed tomography.

Abdominal computed tomography (CT) revealed liver cirrhosis, with a 7.25 × 4.96 cm oval mixed slightly hypodense lesion in the right lobe of an undetermined nature, gallbladder stones, and a left renal cyst (Fig. 1A). Abdominal ultrasound shows liver cirrhosis, with oval lesions measuring 5.5 × 3.7 × 5.6 cm in segment S7 and 2.2 × 2.0 × 1.8 cm at the inferior margin of the left lobe, suggesting a tendency towards malignancy. Multiple mid-to-high echogenic nodules were observed in the remaining liver, the largest measuring 1.0 × 0.9 cm (Fig. 1B). Liver angiography indicates cirrhosis, with lesions in segment S7 (5.6 × 5.5 × 3.7 cm) and the left lobe nodule (2.2 × 2.0 × 1.8 cm) showing no significant blood flow signals, suggesting atypical hemangiomas. Multiple mid-to-high echogenic proliferative nodules were observed in the remaining liver tissues (Fig. 1C). Intraoperative ultrasonography showed that the S7 segment lesion was likely benign (Fig. 1D).

### 2.3. Laboratory tests

Blood routine, liver function and alpha-fetoprotein were normal.

### 2.4. Therapeutic intervention

Due to inconsistent radiological interpretations regarding the nature of the liver mass and given that the patient had hepatitis B-related cirrhosis with a high risk of developing HCC, the attending physician thoroughly discussed the situation with the patient and their family. Surgery was recommended to remove the mass and determine its nature, while also explaining that the mass could potentially be either an HCC or a benign lesion. The patient and their family consented to undergo surgery, and the patient ultimately underwent partial resection of the liver mass.

### 2.5. Pathologic diagnosis

Hepatitis B-associated cirrhosis combined with MNFIL.

### 2.6. Patient follow-up

The patient was followed up for 5 months and is currently doing well.

### 2.7. Diagnostic and treatment process

Given the complexity of the diagnostic and treatment processes in this case, we outlined the clinical course in detail in Table 1.

**Table 1:** Diagnostic and treatment process.

1	Medical examination: CT scan revealed cirrhosis with multiple masses in the liver.
2	Hospitalization for examination:
	CT scan: Liver cirrhosis with live masse, nature of the mass was unclear.
	Ultrasound: Liver cirrhosis with multiple liver masses, masses were suspected to be malignant.
	Hepatic angiography: Liver cirrhosis with multiple liver masses, masses were suspected to be hemangioma.
	Intraoperative ultrasound: Liver cirrhosis with multiple liver masses, masses were suspected to be benign.
3	The patient underwent a partial hepatectomy.
4	Pathological examination: Some physicians supported HCC, while others supported benign findings. Consultation at a higher-level hospital was recommended.
5	The higher-level hospital’s pathology consultation suggested HCC.
6	We requested a second review by 2 experienced radiologists. they all concluded that the masses were benign and suggest they were fatty nodules.
7	We re-examined the histopathological sections and analyzed the immunohistochemical results, the final pathological diagnosis was hepatitis B-associated cirrhosis combined with MNFIL.

CT = computed tomography, HCC = hepatocellular carcinoma, MNFIL = multinodular fatty infiltration.

## 3. Discussion

MNFIL is clinically rare and easily confused with neoplasm, and its diagnosis by imaging and pathology is challenging.^[4]^ In our case, MNFIL occurred in the setting of hepatitis B cirrhosis and was highly susceptible to HCC misdiagnosis. The 2 ultrasound findings were inconsistent, with one tending to indicate a benign condition. Hepatography also suggested benignity, indicating that imaging is valuable for the diagnosis of this disease. However, it still poses diagnostic difficulties for inexperienced physicians, especially in cases of cirrhosis with multiple masses, which are more likely to be misdiagnosed as malignant lesions.

**Figure 2.**
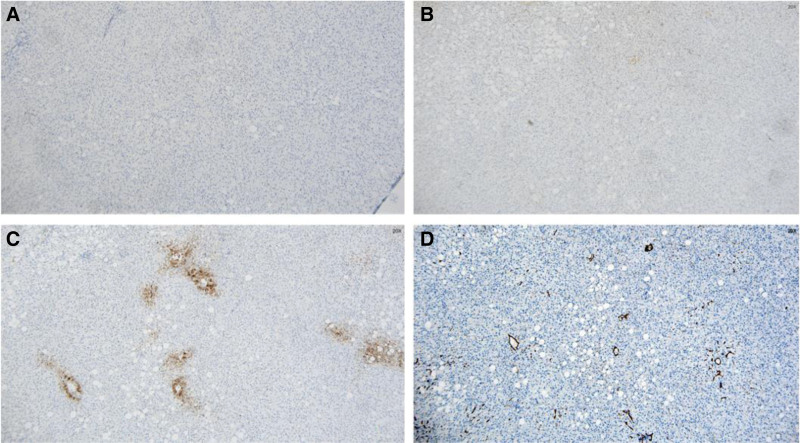
Immunohistochemical expression in MNFIL. (A) The GPC-3 protein expression is negative (IHC, ×4 magnification). (B) HSP70 protein expression is negative (IHC, ×4 magnification). (C) GS protein shows focal positivity (IHC, ×10 magnification). (D) CD34 does not demonstrate diffuse hepatic sinusoidal capillarization (IHC, ×10 magnification). CD34 = cluster of differentiation 34, GPC-3 = glypican-3, GS = glutamine synthetase, HSP70 = heat shock protein 70, IHC = immunohistochemistry, MNFIL = multinodular fatty infiltration.

**Figure 3.**
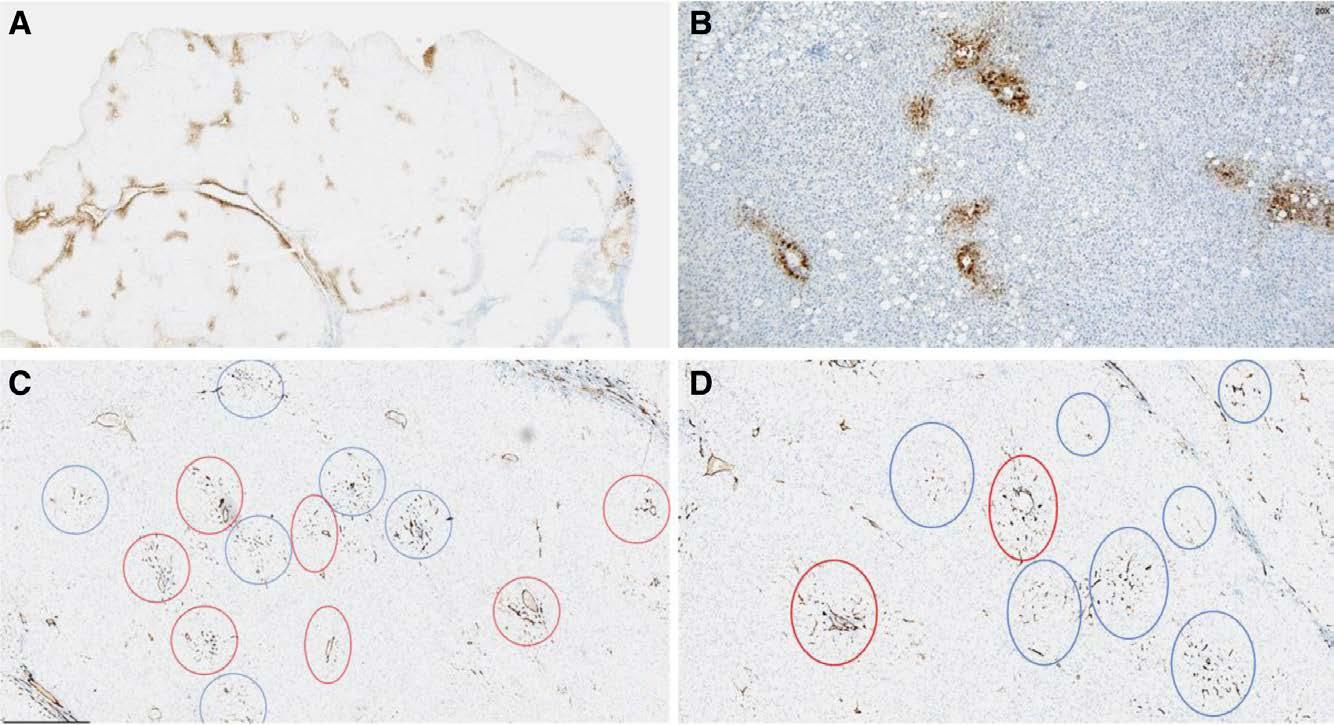
Expression pattern of GS and CD34 protein in MNFIL. (A) GS protein is expressed in hepatocytes around the central vein (IHC, ×1 magnification). (B) GS protein is expressed in hepatocytes around the central vein (IHC, ×10 magnification). (C and D) CD34 shows perisinusoidal fibrosis around the portal area (IHC, ×4 magnification and ×10 magnification). The red circle indicates the presence of blood vessels and bile ducts within the portal area, while the blue circle shows the disappearance of blood vessels and bile ducts within the portal area. CD34 = cluster of differentiation 34, GS = glutamine synthetase, IHC = immunohistochemistry, MNFIL = multinodular fatty infiltration.

**Figure 4.**
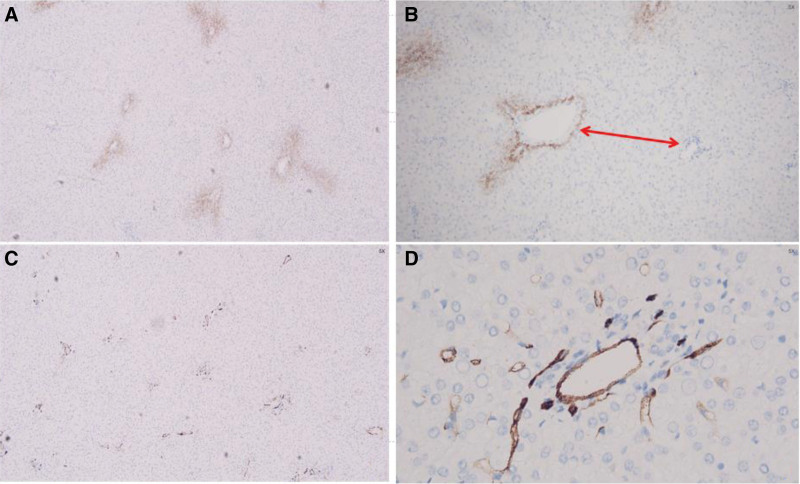
Expression pattern of GS and CD34 protein in normal liver lobules. (A) GS protein is expressed in hepatocytes around the central vein (IHC, ×4 magnification). (B) GS protein is expressed in hepatocytes around the central vein (IHC, ×20 magnification). (C) CD34 shows perisinusoidal fibrosis around the portal area (IHC, ×2 magnification). (D) CD34 shows perisinusoidal fibrosis around the portal area (IHC, ×40 magnification). CD34 = cluster of differentiation 34, GS = glutamine synthetase, IHC = immunohistochemistry.

**Figure 5.**
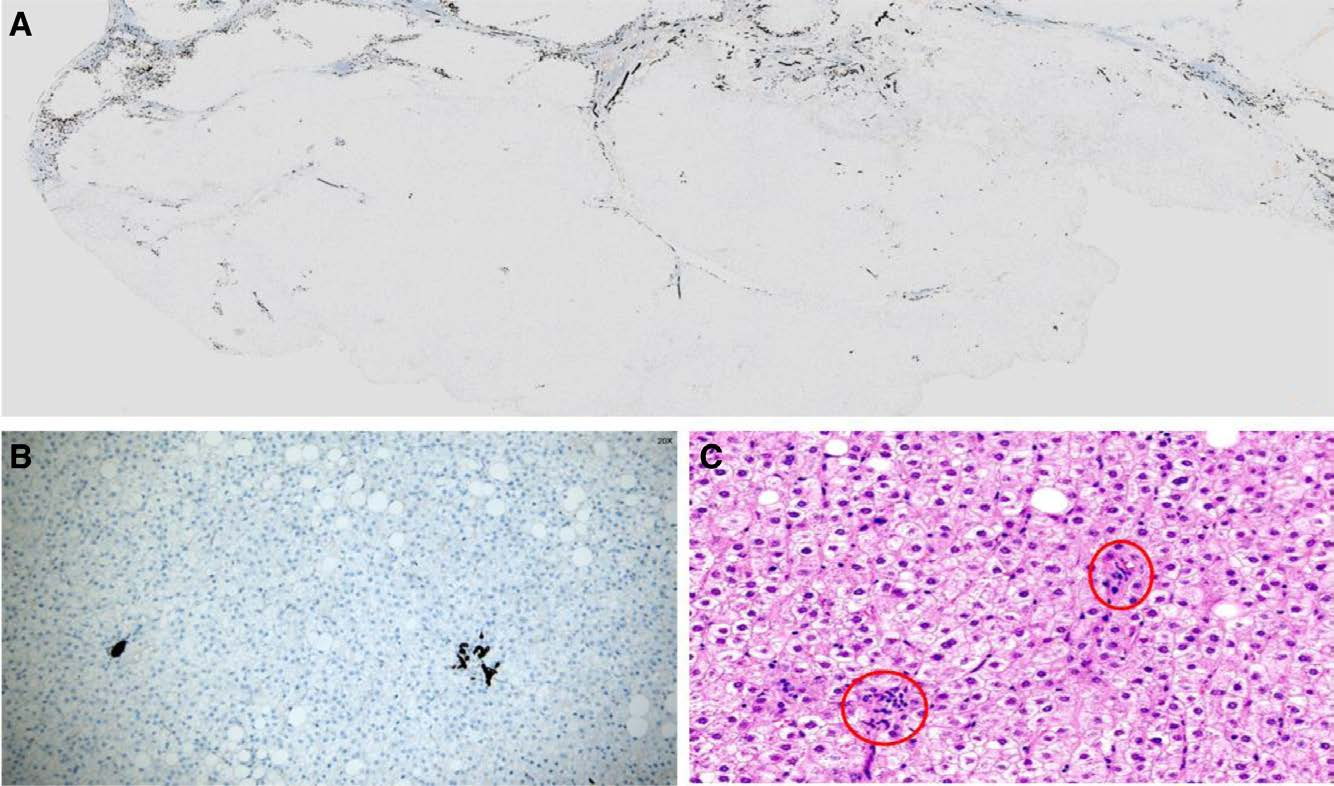
Expression pattern of CK7 protein in MNFIL. (A) Positive CK7 expression around the nodules, indicating the presence of portal areas, while the positivity gradually decreases at the center of the nodules, suggesting the progressive disappearance of bile ducts (IHC, ×1 magnification). (B) CK7 is expressed in residual bile ducts and the corresponding portal vein area is smaller (IHC, ×10 magnification). (C) The residual bile ducts (HE, ×20 magnification). IHC = immunohistochemistry, MNFIL = multinodular fatty infiltration.

Our patient underwent surgical resection and pathologic examination due to inconsistent imaging findings. Pathomorphology was suggestive of HCC, but immunohistochemistry showed negative results for glypican-3 and heat shock protein 70, focally positive results for glutamine synthetase (GS), and cluster of differentiation 34 (CD34) did not demonstrate diffuse hepatic sinusoidal capillarization (Fig. 2). Therefore, some physicians believed that there was insufficient basis for the diagnosis of HCC. We recommend that the higher-level hospital conduct a pathological slide consultation. The consultation concluded that the disease was HCC. Subsequently, we consulted 2 senior imaging physicians, all of them concluded that the lesion was benign and considered fatty infiltration as a possibility. Through in-depth analysis of the immunohistochemical results, we were surprised to find that GS and CD34 were regularly distributed within the nodules, and GS and CD34 expression mimicked the normal hepatic lobular architecture (Fig. 3). In normal liver tissue, GS was expressed in hepatocytes around the central vein and CD34 showed perisinusoidal fibrosis around the portal area (Fig. 4). We carefully analyzed why the morphology could not identify the lobular structures in the liver. We found that the portal area of MNFIL was atrophic, and the veins, arterioles, and bile ducts within the portal area were essentially atrophic and absent (Fig. 3C and D), or only isolated bile ducts were left (Fig. 5). This phenomenon is more pronounced in the center of the nodule. The expression pattern of CD34 confirmed the presence of the portal area, but it was difficult to identify the bile ducts, veins, and arterioles within it using conventional morphology.

**Figure 6.**
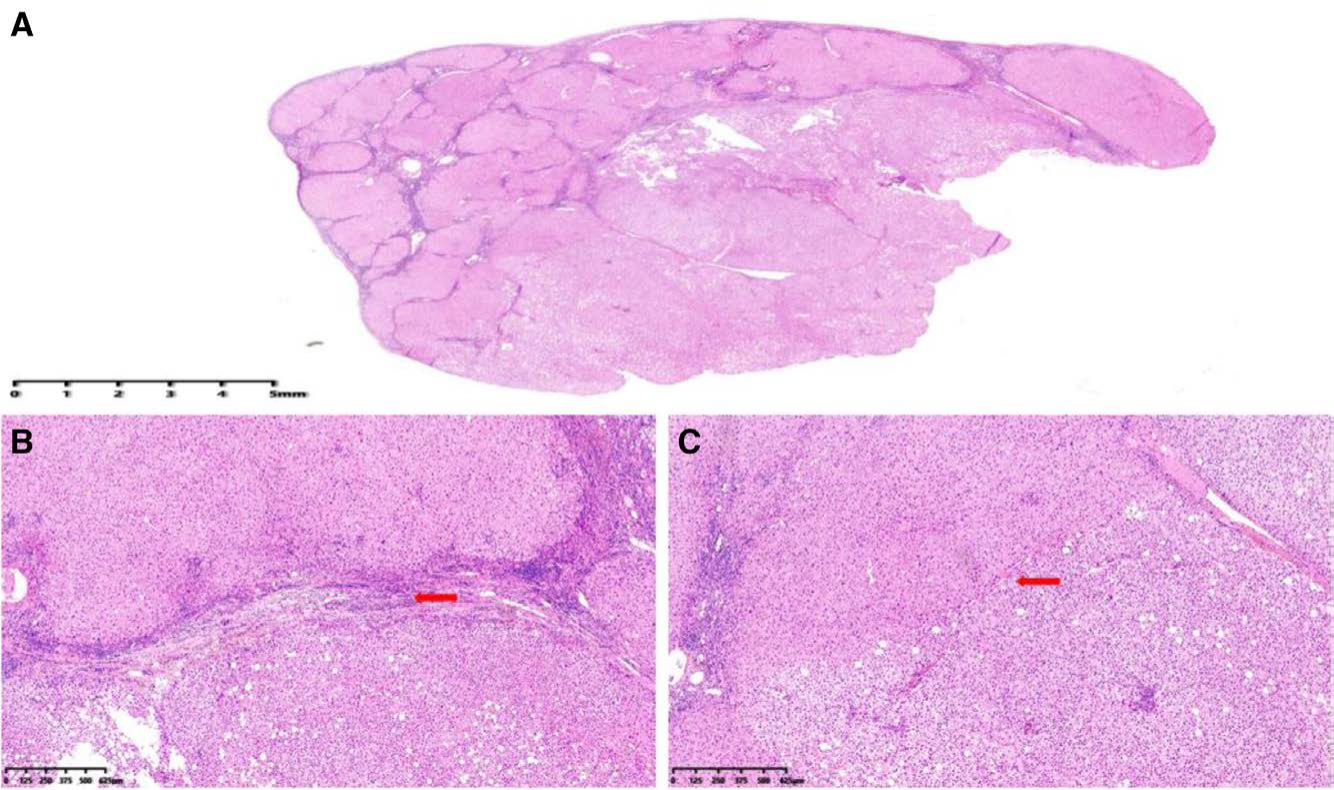
The relationship between MNFIL and the surrounding liver tissue. (A) The relationship between MNFIL and the surrounding liver tissue in this case (HE, ×1 magnification). (B) A clear boundary between the nodule and the surrounding liver tissue (HE, ×4 magnification), while (C) illustrates the fusion between the nodule and the surrounding liver tissue (HE, ×4 magnification). MNFIL = multinodular fatty infiltration.

**Figure 7.**
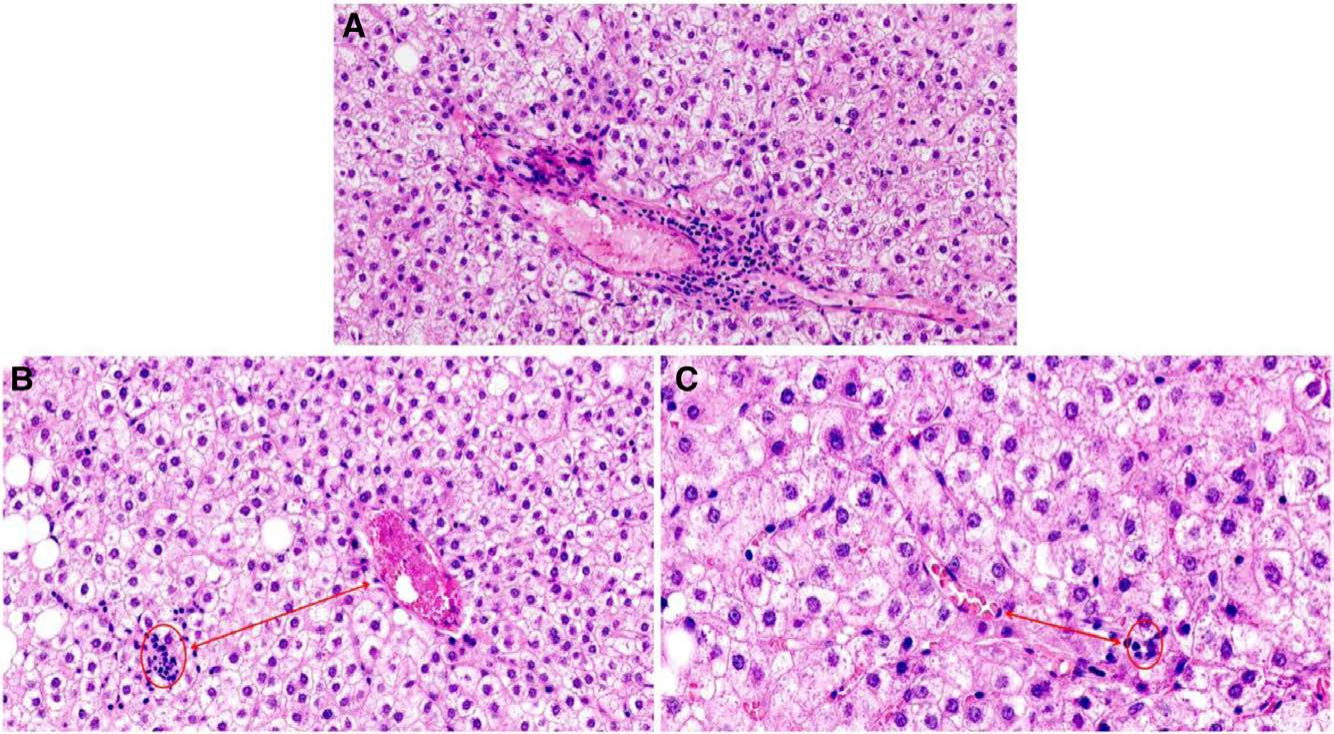
The changes of portal areas in the MNFIL. (A) A small number of morphologically recognizable portal areas are seen in the periphery of the nodule (HE, ×10 magnification). (B, C) The portal areas in the center of the nodule gradually disappear or are difficult to recognize, the red circle shows the residual bile ducts (HE, ×10 magnification). MNFIL = multinodular fatty infiltration.

**Figure 8.**
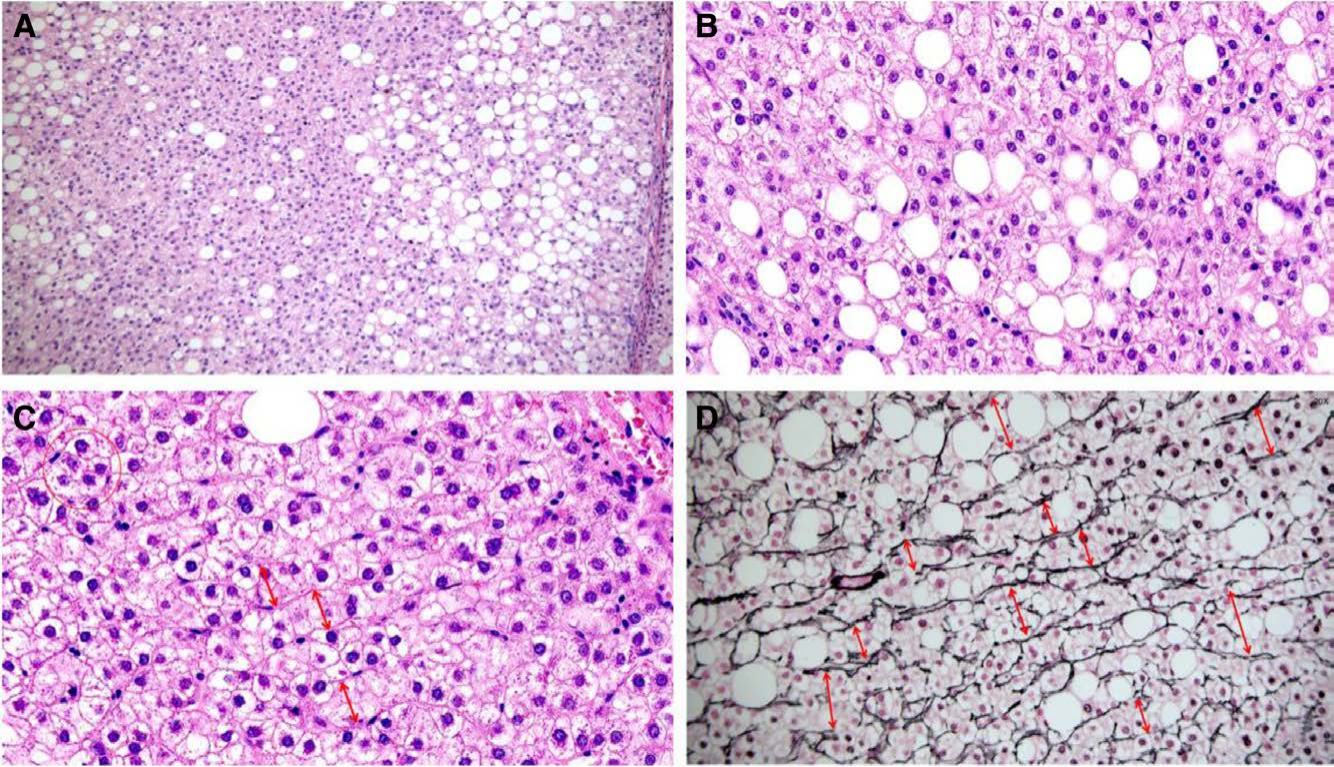
Histological morphology of MNFIL. (A) Shows varying degrees of steatosis within the nodule (HE, ×10 magnification). (B) Hepatocytes exhibit a lack of tumor atypia, and some hepatocytes appear cytoplasmically sparse and exhibit edema (HE, ×20 magnification). (C) Demonstrates varying degrees of thickening in the hepatic plates, with some exceeding 2 layers (HE, ×20 magnification). (D) Reticular fibers indicate thickening of the hepatic plates (special staining, ×20 magnification). MNFIL = multinodular fatty infiltration.

Through analysis of this case, we found that imaging has an absolute advantage in the diagnosis of MNFIL because MNFIL imaging has characteristic changes that are similar to hepatic steatosis, that is, hyperechoic on ultrasonography, hypointense on non-contrast-enhanced CT images, and hypointense on contralesional T1-weighted magnetic resonance imaging and fat-suppressed magnetic resonance imaging sequences.^[2,5,6]^ Searching the literature, we found that MNFIL-related articles were published by imaging specialists or surgeons.^[3,5,7–11]^ All these articles briefly described the presence of steatosis in the morphology of MNFIL, and only a few provided pathological pictures.^[4,5]^ However, none of these studies described the internal structural changes in MNFIL in detail. Based on the morphology of MNFIL in this case, we propose that the pathomorphology of MNFIL can present with the following changes: multiple hyperplastic nodules were identified in the hepatic tissue. Some of these nodules were clearly demarcated from the surrounding tissue, whereas others were fused with it (Fig. 6). A small number of morphologically recognizable portal areas were observed in the periphery of the nodule, whereas the portal areas in the center of the nodule gradually disappeared or were difficult to recognize (Fig. 7). Although most bile ducts and blood vessels in the portal areas were atrophic and absent, immunohistochemistry for GS and CD34 suggested the presence of a lobular structure in the nodules. The hepatic plates were thickened to varying degrees, with some exceeding 2 layers. The degree of steatosis within the nodules varied, and the hepatocytes exhibited a lack of tumor atypia, whereas some hepatocytes showed cytoplasmic edema (Fig. 8).

Pathology has unfavorable factors in the diagnosis of MNFIL, I think there may be the following reasons. First, MNFIL is rare and pathologists have insufficient understanding of the pathological morphology of MNFIL. Second, when pathologists observe steatosis nodules and have no clear portal areas in morphology, they are prone to misdiagnosis as neoplastic lesions such as steatohepatitis subtype HCC and HNF1α inactivated hepatocellular adenoma.^[12,13]^ By recognizing this case of MNFIL, I believe that pathologists can avoid misdiagnosis. When encountering nodules with multiple hepatic steatosis, if glypican-3 and heat shock protein 70 are negative, and CD34 does not show diffuse hepatic sinusoidal capillarization, this is an opportune moment to carefully evaluate the expression patterns of GS and CD34. MNFIL should be considered if GS and CD34 show the presence of the central vein and portal areas, respectively, within the nodule.

**Figure 9.**
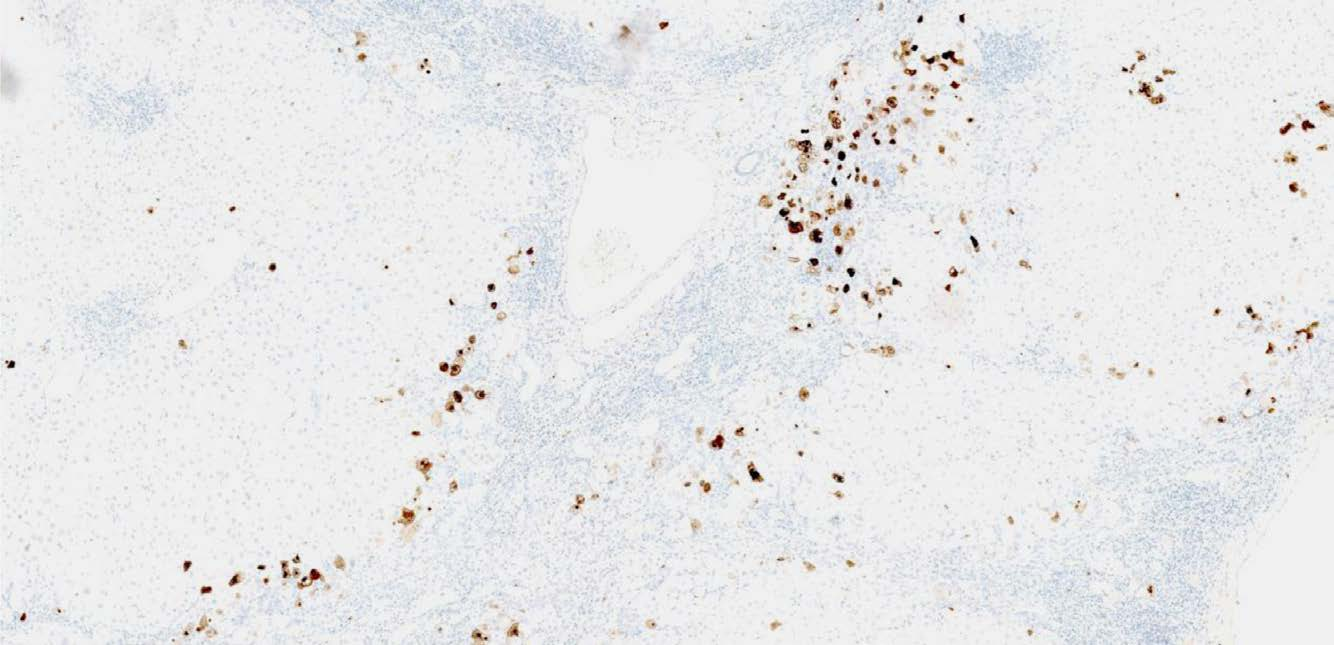
HBsAg protein is expressed in nodular cirrhosis. HBsAg protein is expressed in nodular cirrhosis, suggesting that nodular cirrhosis is caused by hepatitis B virus infection (IHC, ×4 magnification). HBsAg = hepatitis B surface antigen, IHC = immunohistochemistry.

In fact, before immunohistochemistry was performed in this case, almost all pathologists diagnosed HCC due to the presence of a background of hepatitis B-associated cirrhosis; however, this preconception led to misdiagnosis. Therefore, in such complex cases, pathologic diagnosis needs to be closely combined with imaging. Currently, case reports of cirrhosis combined with MNFIL are rare, and cases of hepatitis B-associated cirrhosis combined with MNFIL have not yet been reported. The development of MNFIL is usually associated with factors such as obesity, diabetes mellitus, alcohol consumption, starvation, steroid therapy, or parenteral nutrition; however, this patient had none of these associated risk factors. Our review of relevant literature revealed that chronic viral hepatitis B can affect the metabolism of glucose, lipids, bile acids, and vitamins in patients.^[14]^ Yang et al^[15]^ showed that fatty acid-binding protein 5 and acyl-CoA-binding protein, which are related to fatty acid metabolism and synthesis, were significantly increased in hepatitis B virus transgenic mice (HBV-Tg mice). Hajjou et al^[16]^ found significant upregulation of genes involved in the lipid biosynthesis pathway in HBV-Tg mice, including retinol-binding protein 1, sterol regulatory element binding protein 2, ATP citrate lyase, and fatty acid synthase. Therefore, we speculate that the MNFIL in this case may have been secondary to hepatitis B virus infection (Fig. 9).

When an occupying lesion appears in the context of cirrhosis, clinical physician may initially suspect an HCC. However, this case serves as a reminder that clinical practice should not be confined to fixed thinking patterns as unique individual cases will always arise. In fact, the patient could have avoided excessive surgical intervention from the outset, as the 2 examinations had already suggested a benign possibility. Nevertheless, 1 imaging result suggested HCC, and hepatitis B-related cirrhosis carries a high risk of developing into HCC. Therefore, after the attending physician thoroughly explained the patient’s condition, the patient and their family consented and actively cooperated with the treatment. Following the workup, the mass was confirmed to be benign. The patient was greatly relieved by this outcome, as the definitive characterization of the lesion brought her peace of mind, a benign result being exactly what everyone hopes for.

Pathological diagnosis is considered the gold standard; however, this case presents a significant challenge. Despite consultation at a higher-level hospital, suggesting HCC, we remain uncertain. For such complex cases, our only recourse was to actively request a second review from experienced radiologists. Fortunately, their consensus opinion was that this likely represented a benign lesion, specifically, a fatty nodule. Their recommendations bolstered our confidence in seeking clues to support a benign diagnosis. After reexamining the pathological sections and conducting a detailed analysis of the immunohistochemistry results, we identified evidence supporting a benign lesion. Through this case, we gained valuable experience, deepened our understanding of MNFIL, and provided the first detailed description of its pathological features. This case also underscores that accurate diagnosis and treatment require close collaboration between the clinical and pathological disciplines. Clinical practice serves as the first line of defense against misdiagnosis, whereas pathology is the final bastion against misdiagnosis.

## 4. Conclusions

The presence of multiple nodules in cirrhosis does not necessarily indicate malignant tumors. Maintaining this awareness during the diagnostic process can significantly reduce the likelihood of misdiagnoses.

## Acknowledgments

We sincerely thank the patient’s family for agreeing to use their clinical data in this article. We thank Paperpal Editor (https://preflight.paperpal.com) for English language revision.

## Author contributions

**Funding acquisition**: Wei Sang, Wei Zhang.

**Resources**: Jian Wang, Yuqi Wang.

**Writing – original draft**: Jiayin Zhao.

**Writing – review & editing**: Wei Sang.
